# 

**Published:** 1891-05-09

**Authors:** 


					rmrm
&
HOMAS'S tiOSPi
mir/CH Haspi.tax.
.a soon Western Infirm am
WITH EXTRA NURSING SUPPLEMENT
Saturday, May 9th, 1891.

				

